# Phase I study of sapanisertib (CB‐228/TAK‐228/MLN0128) in combination with ziv‐aflibercept in patients with advanced solid tumors

**DOI:** 10.1002/cam4.6877

**Published:** 2024-02-24

**Authors:** Niamh Coleman, Bettzy Stephen, Siqing Fu, Daniel Karp, Vivek Subbiah, Jordi Rodon Ahnert, Sarina A. Piha‐Paul, John Wright, Senait N. Fessahaye, Fengying Ouyang, Bulent Yilmaz, Funda Meric‐Bernstam, Aung Naing

**Affiliations:** ^1^ Department of Investigational Cancer Therapeutics The University of Texas MD Anderson Cancer Center Houston Texas USA; ^2^ Early Phase Drug Development Sarah Cannon Research Institute Nashville Tennessee USA; ^3^ National Cancer Institute (NCI), Cancer Therapy Evaluation Program (CTEP) Bethesda Maryland USA; ^4^ Khalifa Institute for Personalized Cancer Therapy MD Anderson Cancer Center Houston Texas USA; ^5^ Department of Surgical Oncology MD Anderson Cancer Center Houston Texas USA; ^6^ Present address: Department of Medical Oncology Trinity St. James' Cancer Institute, St. James's Hospital Trinity College Medicine Dublin Ireland

**Keywords:** advanced/refractory cancer, mTOR, mTORC1/2, sapanisertib, targeted therapy, VEGF

## Abstract

**Background:**

Sapanisertib is a potent ATP‐competitive, dual inhibitor of mTORC1/2. Ziv‐aflibercept is a recombinant fusion protein comprising human VEGF receptor extracellular domains fused to human immunoglobulin G1. HIF‐1α inhibition in combination with anti‐angiogenic therapy is a promising anti‐tumor strategy. This Phase 1 dose‐escalation/expansion study assessed safety/ tolerability of sapanisertib in combination with ziv‐aflibercept in advanced solid tumors.

**Methods:**

Fifty‐five patients with heavily pre‐treated advanced metastatic solid tumors resistant or refractory to standard treatment received treatment on a range of dose levels.

**Results:**

Fifty‐five patients were enrolled and treated across a range of dose levels. Forty were female (73%), median age was 62 (range: 21–79), and ECOG PS was 0 (9, 16%) or 1 (46, 84%). Most common tumor types included ovarian (8), colorectal (8), sarcoma (8), breast (3), cervical (4), and endometrial (4). Median number of prior lines of therapy was 4 (range 2–11). Sapanisertib 4 mg orally 3 days on and 4 days off plus 3 mg/kg ziv‐aflibercept IV every 2 weeks on a 28‐day cycle was defined as the maximum tolerated dose. Most frequent treatment‐related grade ≥2 adverse events included hypertension, fatigue, anorexia, hypertriglyceridemia, diarrhea, nausea, mucositis, and serum lipase increase. There were no grade 5 events. In patients with evaluable disease (*n* = 50), 37 patients (74%) achieved stable disease (SD) as best response, two patients (4%) achieved a confirmed partial response (PR); disease control rate (DCR) (CR + SD + PR) was 78%.

**Conclusion:**

The combination of sapanisertib and ziv‐aflibercept was generally tolerable and demonstrated anti‐tumor activity in heavily pre‐treated patients with advanced malignancies.

## Graphical abstract

The AKT pathway is frequently dysregulated in human cancer. This Phase 1 dose‐escalation/expansion study assessed safety/ tolerability of sapanisertib in combination with ziv‐aflibercept in advanced solid tumors. The combination of sapanisertib and ziv‐aflibercept was generally tolerable and demonstrated anti‐tumor activity in heavily pre‐treated patients with advanced malignancies.

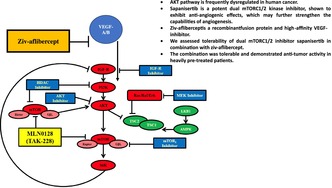

## 1 INTRODUCTION

The phosphoinositide 3‐kinase (PI3K)/protein kinase B (AKT)/mammalian target of rapamycin (mTOR) signaling pathway is one of the most frequently deregulated pathways in human cancer and a key regulator of cellular proliferation, growth, and survival.^1^ Pathway activation occurs in several ways at various levels of the pathway, such as by mutation or upregulation of upstream proteins (such as *PI3K* or *AKT*).^2^ *PI3K/AKT* pathway aberrations have been identified in almost 40% of all solid tumors and *PTEN* loss by IHC occurs most frequently (30%), followed by mutations in *PIK3CA* (13%), *PTEN* (6%), and *AKT* (1%).^3^ Dysregulation of the pathway is associated with the development and maintenance of numerous solid tumors, including lung, breast, head and neck, ovary and colon cancer.^4^, ^5^, ^6^, ^7^, ^8^, ^9^, ^10^ Consequently, targeting this pathway has been the focus of oncology trials for many decades.

mTOR is a serine–threonine kinase and a key intracellular point of convergence for several pathways in human cancer and thus represents an important therapeutic target. mTOR exists as two complexes, one with raptor, mTOR complex 1 (mTORC1) which is rapamycin‐sensitive, and the other with rictor, which is typically rapamycin insensitive (mTORC2).^11^ mTOR complex 1 (mTORC1) phosphorylates 4EPB1 and p70S6 kinase and results in translation of proteins involved in cell cycle progression, while rapamycin‐insensitive mTORC2 has been shown to directly phosphorylate and activate AKT at serine 473.^12^ Inhibition of mTORC1 inhibits the negative feedback loop between S6 kinase and insulin receptor substrate which results in an increase in *PI3K* and *AKT* activity which may limit the activity of rapamycin and impair rapalog efficacy.^13^, ^14^

Rapamycin analogues, such as everolimus, exert their effect predominantly on mTORC1, with minimal inhibitory effect on mTORC2, and have been approved for the treatment of advanced renal cell cancer, breast cancer, and other solid tumors.^15^, ^16^, ^17^ Sapanisertib (formerly TAK‐228 or MLN0128) is a potent, selective, oral dual inhibitor of mTORC1 and TORC2, developed to address incomplete inhibition of mTOR by rapalogs. Pre‐clinical models support the potency of this dual inhibition of mTORC1 and mTORC2 strategy.^18^, ^19^, ^20^, ^21^ In addition, recently published early phase clinical trials have demonstrated promising anti‐tumor activity and manageable safety profile of single agent sapanisertib in endometrial and renal cell carcinoma.^22^

Angiogenesis has been implicated in tumor development and metastasis,^23^ and is partly mediated by vascular endothelial growth factor (VEGF).^24^ The anti‐angiogenic properties of mTOR inhibitors have been well documented in both in vitro and in vivo models.^25^, ^26^ There are pre‐clinical data to suggest that hypoxia‐inducible factor 1 α (HIF‐1α) is modulated by mTORC1,^27^ and that mTORC1 drives HIF‐1α and VEGF‐A signaling via multiple signaling mechanisms involving 4E‐BP1, S6K1, and STAT3.^27^ Furthermore, STAT3 has been shown to be directly phosphorylated by mTORC1 on Ser727 during hypoxia, promoting HIF‐1α mRNA transcription. Increased levels of HIF‐1α have been associated with increased expression of VEGF, aggressive tumor growth, and poor patient prognosis.^13^ This phenomenon has been observed as a mechanism of resistance in tumors treated with anti‐VEGF therapy.^28^ mTOR pathway inhibition together with VEGFR pathway inhibition has shown synergism in renal cell carcinoma using pre‐clinical models.^29^ HIF‐1α inhibition in combination with anti‐angiogenic therapy may further strengthen the capabilities of angiogenesis inhibitors^30^ and is a promising strategy for targeting tumor resistance.^28^, ^31^

Ziv‐aflibercept is a recombinant fusion protein consisting of human VEGF receptor extracellular domains fused to the Fc portion of human immunoglobulin G1 (IgG1) and contains portions of the extracellular domains of 2 different VEGFRs: VEGFR1 (also known as Flt‐1) and VEGFR2 (also known as KDR or Flk‐1). mTOR pathway inhibitors, such as sapanisertib, inhibit the activity of several angiogenic factors, including HIF‐1α, which result in decreased VEGF and decreased angiogenic activity.^31^ Moreover, a phase III study of everolimus in pancreatic neuroendocrine tumors demonstrated that mTOR inhibition may reduce circulating levels of sVEGFR1, PlGF, and bFGF.^32^

Altogether, these data suggested that sapanisertib would be a suitable candidate for combination therapy with ziv‐aflibercept and we hypothesized that combination therapy could enhance anti‐tumor activity of sapanisertib and target tumor resistance. Here, we report the preliminary safety, tolerability, and efficacy from the dose‐escalation study of sapanisertib in combination with aflibercept in patients with advanced solid tumors.

## 2 METHODS

### 2.1 Study design

This was an open‐label, single‐center Phase I clinical trial that employed a 3 + 3 dose‐escalation design and was conducted at The University of Texas M.D. Anderson Cancer Center and supported by NCI‐CTEP. The primary endpoint was to evaluate the safety and tolerability of sapanisertib in combination with ziv‐aflibercept, to determine maximum tolerated dose (MTD) and dose‐limiting toxicities (DLT) of the combination in patients with advanced cancers refractory to standard therapy. Secondary objectives included the evaluation of preliminary anti‐tumor efficacy of the combination treatment per response evaluation criteria in solid tumors (RECIST version 1.1)^33^ and to evaluate AKT/mTOR signaling and adaptive responses.

One cycle consisted of 4 weeks of treatment (28 days), with sapanisertib taken orally for 3 days on and 4 days off starting on Cycle 1 Day 2 (and starting Day 2 of every cycle). Ziv‐aflibercept was given via intravenously (IV) infusion once every 2 weeks (Days 1 and 15 for each cycle). Both study drugs were permitted a +/− 2 day window, though not permitted to be given on the same day.

Trial enrolment was challenging due to need to re‐start the dose escalation three times, which included modification to the dosing schedule, and drug formulation changes. There were three original planned dose escalations scheduled (Table S1). These dose levels were subsequently modified, and patients were treated in the dose levels as shown in Table 3. During dose‐escalation, patients received one of four sapanisertib doses (3 mg/ 4 mg/ 5 mg / 6 mg) once daily (QD) for 3 days on/4 days off in combination with ziv‐aflibercept in one of three doses (2, 3 or 4 mg/kg) given intravenously IV every 2 weeks, in a 28‐day cycle (Table 3). Once the MTD was determined, the expansion cohort was opened (Table S2). Tumor measurements were performed every 8 weeks. Data cut‐off date was December 15, 2021. There were no planned intra‐patient dose escalation, and no patients were enrolled in the next dose level until the toxicity was fully assessed following completion of 1 cycle and at least 3 patients enrolled at the previous dose level.

Per study protocol, DLTs were defined as an adverse events (AE) occurring within the first cycle deemed related to study agents with an attribution of possible, probably, or definite and fulfilling one of the following criteria: grade 4 neutropenia lasting >7 days, febrile neutropenia (defined as absolute neutrophil count [ANC] <1.0 × 10^9^/L and fever ≥38.5°C) or documented grade ≥3 infection with ANC ≤1.0 × 10^9^/L, platelet count <25,000/mm lasting >7 days, and any grade ≥3 non‐hematologic toxicity that persists for >7 days, except for the following: nausea/vomiting, diarrhea, and electrolyte imbalances; grade 3 laboratory abnormalities that are asymptomatic and responsive to supportive measures and that are without clinical consequence; grade 3 hyperglycemia or grade 3 diabetes that can be stably controlled; grade 3 laboratory abnormalities that are asymptomatic and responsive to supportive measures and were without clinical consequence; and grade 3 hypertension that resolved within 14 days with medical management. Patients who experienced proteinuria that resolved to <2 g within 14 days were not defined as a DLT. Hypersensitivity/Allergic reactions with expected severity and presentation were not considered a DLT. Patients who withdrew from the study prior to completion of the first cycle of study treatments for reasons other than treatment‐related AEs were replaced.

### 2.2 Patients

All patients provided written informed consent for participation. Eligible patients included patients with metastatic or advanced solid tumor resistant or refractory to standard therapy, an Eastern Cooperative Oncology Group (ECOG) performance status 0 to 1 and adequate hematologic, hepatic, and renal function. Patients with known brain metastases were excluded from the study. Patients had to have the ability to swallow oral medications and patients with uncontrolled diabetes mellitus (defined as fasting serum glucose >130 mg/dL despite best medical management or HbA1c >7%) and uncontrolled hypertension (defined as blood pressure >150/95 mmHg, or systolic blood pressure >180 mmHg when diastolic blood pressure <90 mmHg, on at least 2 repeated determinations on separate days within 3 months prior to study enrolment) were excluded. Prior treatment with mTOR inhibitors, TORC1/2 inhibitors, TORC1 inhibitors, or AKT inhibitors was permitted. Patients with history of significant cardiovascular or pulmonary disease, intercurrent uncontrolled illness, malabsorption due to prior gastrointestinal (GI) surgery, and GI disease (eg. patients with enteric stomata were excluded) were excluded. Urine protein screen by dipstick or urine analysis was required; for proteinuria >1+ or urine protein: creatinine ratio >1.0, 24‐hour urine protein was recommended to be obtained and level of <2000 mg was required for patient enrollment. Patients were permitted to have evaluable or measurable disease by RECIST v1.1.^33^

### 2.3 Assessments

Data from all patients who received one or more doses of drug were incorporated into the final safety analysis. All patients who receive any amount of the study drug(s) were evaluable for toxicity. The MTD was defined as the highest dose level at which no more than 1 of 6 evaluable patients experienced a DLT. If multiple toxicities were seen, the presence of DLT was based on the most severe toxicity experienced. The response‐evaluable population was defined as all patients who had measurable disease at baseline according to RECIST version 1.1, who had received at least 1 dose of any study drug, and who had at least 1 available post‐baseline response assessment as per RECIST version 1.1. Response was assessed according to the RECIST v1.1 following every two cycles of treatment. AEs were assessed using the National Cancer Institute Common Terminology Criteria for Adverse Events, (NCI CTCAE version 4.0 until March 31, 2018, and version 5.0 beginning April 1, 2018). Detailed descriptions of predefined DLTs, management of AEs, and safety and efficacy assessments are included in the Protocol.

### 2.4 Statistical analysis

No formal hypotheses were tested, and analyses were descriptive and exploratory.

## 3 RESULTS

### 3.1 Patients

Fifty‐five patients with advanced or metastatic solid tumors were enrolled and treated with sapanisertib and ziv‐aflibercept at a range of doses (Table 3). Seventy‐three percent of patients were female (*n* = 40), and the overall median age was 62 (range 21–79 years) (Table 1). The most frequent tumor types enrolled were ovarian (*n* = 8, 15%), colorectal (*n* = 8, 15%), sarcoma (*n* = 8, 15%), endometrial (*n* = 4, 7%), cervical (*n* = 4, 7%), breast (*n* = 3, 5%), hepatocellular (*n* = 3, 5%), and neuroendocrine (*n* = 3, 5%). Most patients were ECOG PS 1 (84%); median number of prior lines of therapy was 4, and most patients had received more than two lines of therapy (72%, *n* = 40) (Table 1).

**TABLE 1:** Baseline patient demographics.

Characteristic	Number (*n* = 55) (%)
Gender	
Female	40 (73)
Male	15 (27)
Median age at study enrollment, years (range)	62 (21–79)
Ethnicity	
White	46 (84)
Black	3 (8)
Hispanic	1 (2)
Unknown	3 (5)
Other	2 (4)
Disease type	
Sarcoma	9 (16)
Ovarian	8 (15)
Colorectal	8 (15)
Endometrial	4 (7)
Cervical	4 (7)
Breast cancer	3 (5)
Hepatocellular	3 (5)
Neuroendocrine	3 (5)
Renal cell	2 (4)
Head and neck SCC	2 (4)
Merkel cell carcinoma	1 (2)
Mesothelioma	1 (2)
NSCLC	1 (2)
Melanoma	1 (2)
Prostate	1 (2)
Cholangiocarcinoma	1 (2)
Vaginal	1 (2)
Cancer of unknown primary	1 (2)
ECOG PS	
0	9 (16)
1	46 (84)
Number of prior therapies (range)	(1–11)
1–2	15 (27)
3–4	19 (35)
>4	21 (38)

### 3.2 Treatment response

**FIGURE 1:**
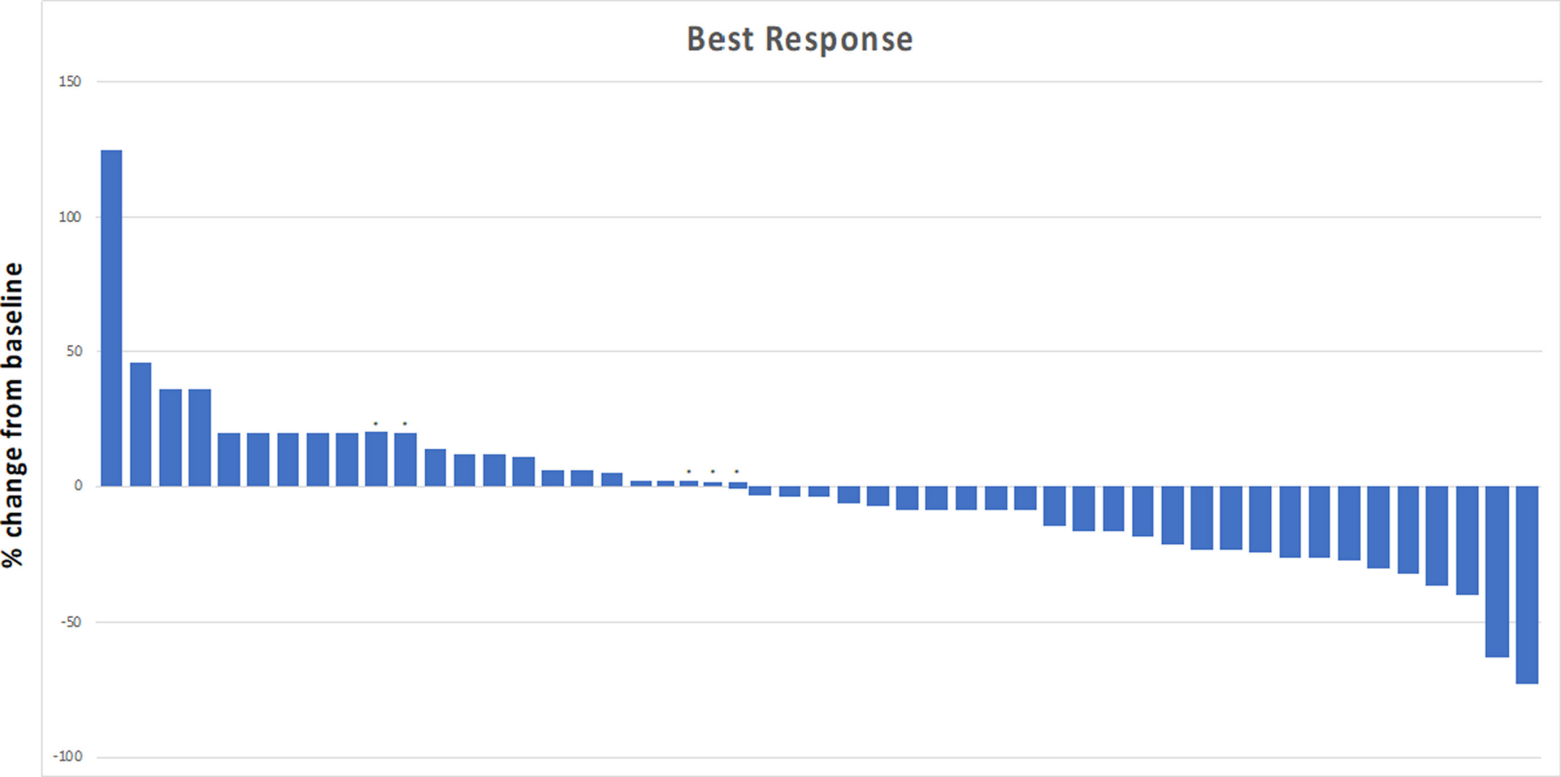
Waterfall plot showing best overall response of evaluable patients on trial. Among 55 patients in the data set, 50 patients were response evaluable, of whom 5 had non‐measurable disease by RECIST v1.1 criteria. Two patients (4%) achieved confirmed partial response (PR), 37 had stable disease (SD) (74%) (including four patients with unconfirmed partial responses); 11 patients (22%) had progression of disease (PD) as best response. Five patients were NE for response. The five patients with non‐measurable, but evaluable disease and are denoted by *; two of these patients had clinical PD, and three of these patients achieved stable disease.

**FIGURE 2:**
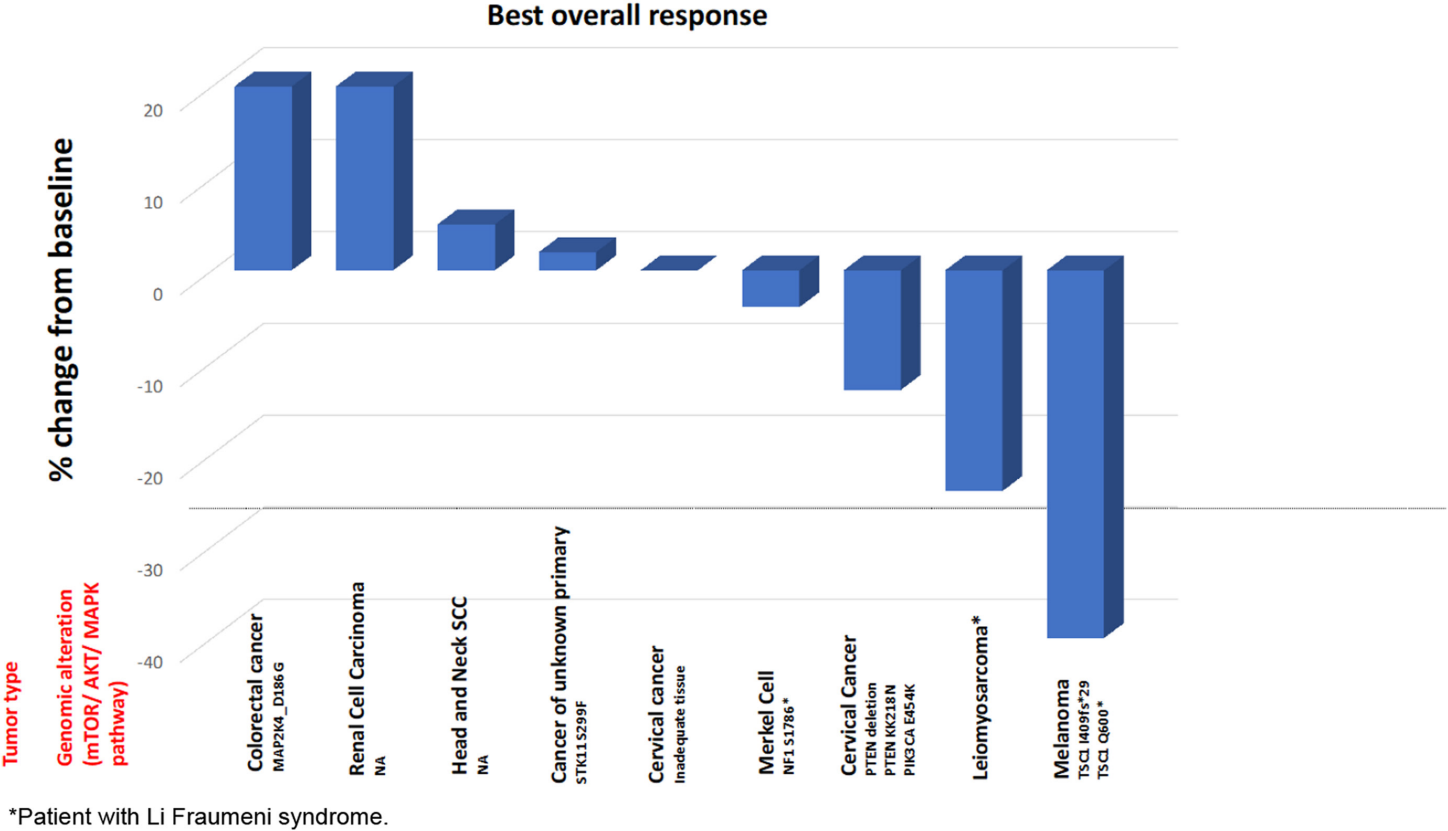
Waterfall plot showing best overall response of evaluable patients in the dose expansion cohort. Ten patients were treated in the dose expansion cohort; among nine evaluable patients, one patient achieved confirmed partial response, five patients had stable disease as best response and three patients had progression of disease (one with clinical progression of disease), arbitrarily assigned 20% increase from baseline measurements). *Patient with Li Fraumeni syndrome.

Overall, there were 50 response‐evaluable patients, including five patients with non‐measurable but evaluable disease. Five patients were not evaluable for response as they came off treatment prior to first re‐staging scan. Of the 50 patients evaluable for response, two patients (4%) had confirmed PR, 37 patients (74%) had SD, including four patients with unconfirmed PR, and 11 patients (22%) had PD, including two patients with clinical progression of disease (Table S3). The overall response rate (ORR) was 4%, and disease control rate (DCR; CR + SD + PR) was 78% (Figure 1). The most frequent reason for study discontinuation was PD in 64% of patients. PRs were achieved at dose level 1 (*n* = 1), dose level 2 (*n* = 1), and dose level 3 (*n* = 2), and these unconfirmed partial responses were observed in patients with leiomyosarcoma (*n* = 2), breast cancer (*n* = 1), and endometrial cancer (*n* = 1), (Figure 2).

The most frequent reason for study discontinuation was progression of disease (PD) in 64% of all patients.

### 3.3 Adverse events

The most frequent treatment‐related AEs (severity graded per NCI CTCAE criteria v5.1) are summarized in Table 2. Forty‐six out of 55 patients (84%) had at least one treatment‐related AE during the study; however, these were predominantly grade 1 or 2 in severity (Table 2). The most common treatment‐related grade ≥2 AEs were hypertension (25%, *n* = 14), fatigue (24%, *n* = 13), anorexia (13%, *n* = 7), hypertriglyceridemia (13%, *n* = 7), diarrhea (11%, *n* = 6), nausea (11%, *n* = 6), mucositis (11%, *n* = 6), and serum lipase increase (11%, *n* = 6). Grade 4 treatment‐related events occurred in three unique patients as follows: maculopapular rash (possibly related to sapanisertib), reversible posterior leukoencephalopathy syndrome (possibly related to ziv‐aflibercept), and lipase increase (possibly related to both study drugs) [*n* = 1, each; 1.8%]. There were no grade 5 treatment‐related AEs (Table 2; Table S4).

**TABLE 2:** Treatment‐related adverse events in all patients receiving sapanisertib in combination with ziv‐aflibercept.

Adverse event	G1	G2	G3	G4	Any grade
Total	131	69	51	3	254
Mucositis oral	18	4	1		23
Fatigue	5	8	5		18
Hypertension	1	5	9		15
Hypertriglyceridemia	6	4	3		13
Nausea	7	2	4		13
Anorexia	3	7			10
Aspartate aminotransferase increased	6	1	2		9
Cholesterol high	8	1			9
Alanine aminotransferase increased	5	3			8
Rash maculopapular	3	2	3		8
Vomiting	2	2	4		8
Headache	5	1	1		7
Hyperglycemia	2	4	1		7
Proteinuria	1	5	1		7
Abdominal pain	2	2	2		6
Diarrhea	3	2	1		6
Platelet count decreased	4	2			6
Creatinine increased	3	1	1		5
Investigations—Other	4	1			5
Epistaxis	3		1		4
Lipase increased		1	2	1	4
Anemia	1	2			3
Gastrointestinal disorders—Other	3				3
Hoarseness	2	1			3
Skin and subcutaneous tissue disorders—Other	2	1			3
Alkaline phosphatase increased	2				2
Arthralgia	2				2
Blood bilirubin increased	2				2
Cardiac disorders—Other (Tachycardia)	1	1			2
Constipation	2				2
Dehydration		1	1		2
Dysgeusia	1	1			2
General disorders and administration site conditions—Other	2				2
Nervous system disorders—Other	2				2
Oral pain	2				2
Pancreatitis			2		2
Rash acneiform	1		1		2
Ascites	1				1
Back pain	1				1
Cough		1			1
Dyspepsia	1				1
Dysphasia			1		1
Dyspnea			1		1
Esophageal pain	1				1
Eye pain	1				1
Fever	1				1
Gastritis			1		1
Hematuria	1				1
Hemoglobin increased	1				1
Hemorrhoids	1				1
Hyperkalemia	1				1
Insomnia	1				1
Metabolism and nutrition disorders—Other	1				1
Musculoskeletal and connective tissue disorder—Other	1				1
Oral dysesthesia	1				1
Osteonecrosis of jaw		1			1
Pain			1		1
Papulopustular rash				1	1
Peripheral sensory neuropathy	1				1
Pruritus		1			1
Pulmonary fibrosis			1		1
Reversible posterior leukoencephalopathy syndrome				1	1
Transient ischemic attacks		1			1
Urinary tract obstruction			1		1
White blood cell decreased	1				1

*Note*: Most common treated‐related adverse events are summarized by severity, severity based on NCI CTCAE criteria version 5.

### 3.4 DLTs and MTD determination

Dose escalation, DLTs, and MTDs are summarized in Table 3. Doses were escalated to modified dose level 6, sapanisertib 6 mg orally 3 days on and 4 days off plus 4 mg/kg ziv‐aflibercept IV every 2 weeks on a 28‐day cycle (Table 3). There were no DLTs reported until this cohort was treated (two DLTs in two separate patients). One patient (1 out of 4) experienced DLT of grade 3 transient ischemic attack (TIA) related to ziv‐aflibercept; the second patient experienced DLT of grade 3 gastritis. Doses were reduced to next dose level (dose level 5), sapanisertib 5 mg orally 3 days on and 4 days off plus 3 mg/kg ziv‐aflibercept IV every 2 weeks on a 28‐day cycle (Table 3), where one patient experienced DLT with grade 3 dyspnea, possibly related to both study drugs. Doses of both study drugs were modified, and the patients were treated in the expansion cohort using sapanisertib 4 mg orally 3 days on and 4 days off plus 3 mg/kg ziv‐aflibercept IV every 2 weeks on a 28‐day cycle. No DLTs observed in the expansion cohort. Based on these findings, sapanisertib 4 mg orally 3 days on and 4 days off plus 3 mg/kg ziv‐aflibercept IV every 2 weeks on a 28‐day cycle was defined as the maximum tolerated dose.

**TABLE 3:** Summary of dose escalations and dose‐limiting toxicities in patients treated on study.

Dose level	MLN0128 (TAK‐228) (mg PO)	Ziv‐Aflibercept (mg/kg IV Q 2 weeks)	Treated patients, *n*	Patients with DLTs, *n*	DLT	Causality	Comments
Level 1	3 QD	3	5	0	0		5 patients received drug here 5 pts treated patients; 2 pts were not DLT evaluable
Level 1	3 (3 days on/4 days off)	2	4	0	0		Dose level interrupted due to formulation change; Note change in ziv‐aflibercept dose level 4 pts treated 1 pt had to be replaced as not given enough drug to meet DLT criteria
Level 2	3 (3 days on/4 days off)	3	3	0	0		
Level 3	4 (3 days on/4 days off)	3	3	0	0		
Level 4	4 (3 days on/4 days off)	4	4	0	0		4 pts treated 1 pt not DLT evaluable
Level 5	5 (3 days on/4 days off)	4	3	0	0		
Level 6	6 (3 days on/4 days off)	4	6	2	Grade 3 TIA Grade 3 gastritis	Related to ziv‐aflibercept Related to both drugs	2 pts were not DLT evaluable Note dose reductions in both drugs following DLTs
Level 5a	5 (3 days on/4 days off)	3	4	1	Grade 3 dyspnea	Possibly related to both drugs	Note this differs from above dose Level 5 due to reduced dose level ziv‐aflibercept 1 patient NE (withdrew consent) 1 DLT 1 pt with poor tolerability at this dose level
Level 4a	4 (3 days on/4 days off)	2	3	0	0		Note this differs from above dose Level 4 due to reduced dose level ziv‐aflibercept Here – doses were well tolerated, ziv‐aflibercept dose increased
Level 2a	4 (3 days on/4 days off)	3	9	0	0		Note this differs from above dose Level 2 due to TAK‐228 dose level 3 pts were not evaluable; therefore, it was decided to ensure safety to add 3 more patients, and then safely move to the dose expansion cohort.
Level 2a expansion	4 (3 days on/4 days off)	3	10	0	0		

Abbreviations: DLT dose‐limiting toxicity; MTD maximum tolerated dose; NE, not evaluable.

## 4 DISCUSSION

This open‐label single‐institution phase I trial confirmed the safety and tolerability of sapanisertib in combination with ziv‐aflibercept in heavily pre‐treated patients with advanced refractory cancer. The safety profile of mTORC1/2 inhibitor sapanisertib in combination with ziv‐aflibercept was generally tolerable, and toxicities observed with both agents were mostly grade 1–2 and consistent with previously published attributions in the literature. Sapanisertib 4 mg orally 3 days on and 4 days off plus 3 mg/kg ziv‐aflibercept IV every 2 weeks on a 28‐day cycle was defined as the maximum tolerated dose and recommended phase II dose. No new safety signals were identified for either agent.

Recently, a phase I study of sapanisertib, a dual mTORC1/2 inhibitor, was conducted in patients with advanced solid tumors with expansion cohorts in renal, endometrial, or bladder cancer.^22^ Here, Voss et al. confirmed the pharmacodynamic effect of sapanisertib on downstream effectors of TORC1 (p4EBP1 and pS6) and TORC2 (pPRAS40 and pNDRG1), with treatment‐related decreases in p4EBP1, pS6, pPRAS40, and pNDRG1 using single agent sapanisertib doses of ≥4 mg.^22^ Other groups have previously demonstrated treatment‐related reductions in mTORC biomarkers, including TORC1/2 skin biomarkers (phosphorylated S6, 4EBP1, and PRAS40),^34^ which support the dual TORC1/2 inhibitory activity of sapanisertib. In another study published this year, Paik et al demonstrated potent activity of TAK‐228 in NSCLC models harboring Nrf2 activating alterations^35^ and confirmed single agent clinical activity in genomically selected squamous cell lung cancer with NFE2L2/KEAP1 alterations, while also suggesting a role for combination therapy in treatment resistant patients.^35^

We demonstrate preliminary anti‐tumor activity using sapanisertib in combination with ziv‐aflibercept across tumor types. While the overall response rate of the combination was muted (4%), most patients derived clinical benefit, with 80% of evaluable patients achieving disease control (SD and/or PR); 37 patients (74%) achieved stable disease. Of the patients with stable disease as best response, four of these patients had initial unconfirmed partial responses. The two patients with unconfirmed PRs on imaging had AKT E17K hotspot mutations, while of the two patients who achieved confirmed partial responses, one patient had dual TSC1 mutations, suggesting a potential role for this combination in patients with activation of the *mTOR/AKT/PI3K pathway*.

There are several limitations of this study. The duration of the study was prolonged due to delayed enrollment because of drug formulation changes and dosing schedule modification. The inclusion of many patients with co‐mutations in addition to *mTOR/AKT/PI3K* pathway alterations also may have confounded the interpretation of overall response to the combination strategy.

In conclusion, we demonstrate that the combination strategy utilizing sapanisertib and ziv‐aflibercept is safe and well tolerated, with some clinical benefit demonstrated in our cohort of heavily pre‐treated patients with and without *mTOR/AKT/PI3K* pathway alterations. Further study of sapanisertib in combination with ziv‐aflibercept may be warranted at the recommended phase II dose of sapanisertib 4 mg orally, 3 days on and 4 days off, plus 3 mg/kg ziv‐aflibercept IV every 2 weeks on a 28‐day cycle. In conclusion, sapanisertib in combination with ziv‐aflibercept has exhibited preliminary anti‐tumor activity and a favorable safety profile in late‐line patients with advanced solid tumors.

## AUTHOR CONTRIBUTIONS

**Niamh Coleman:** Data curation; formal analysis; methodology; validation; writing – original draft; writing – review and editing. **Bettzy Stephen:** Conceptualization; data curation; formal analysis; funding acquisition; investigation; methodology; project administration; resources; software; supervision; validation; visualization; writing – original draft; writing – review and editing. **Siqing Fu:** Project administration; resources; supervision; writing – review and editing. **Daniel Karp:** Project administration; resources; supervision; writing – review and editing. **Vivek Subbiah:** Methodology; project administration; supervision; writing – review and editing. **Jordi Rodon Ahnert:** Investigation; methodology; project administration; supervision; writing – review and editing. **Sarina A. Piha‐Paul:** Investigation; methodology; project administration; supervision; writing – review and editing. **John Wright:** Conceptualization; data curation; formal analysis; funding acquisition; investigation; methodology; project administration; resources; software; supervision; validation; visualization; writing – original draft; writing – review and editing. **Senait N. Fessahaye:** Investigation; methodology; project administration; resources; software; supervision; writing – review and editing. **Fengying Ouyang:** Investigation; methodology; project administration; resources; supervision; writing – review and editing. **Bulent Yilmaz:** Investigation; methodology; project administration; writing – review and editing. **Funda Meric‐Bernstam:** Conceptualization; data curation; formal analysis; funding acquisition; investigation; methodology; project administration; resources; software; supervision; validation; visualization; writing – original draft; writing – review and editing. **Aung Naing:** Conceptualization; data curation; formal analysis; funding acquisition; investigation; methodology; project administration; resources; software; supervision; validation; visualization; writing – original draft; writing – review and editing.

## FUNDING INFORMATION

National Cancer Institute (NCI) CTEP, and MD Anderson Cancer Center.

## CONFLICT OF INTEREST STATEMENT

NC, BS, SF, FO, BY, FS, and JW declare no conflicts of interest. JR reports non‐financial support and reasonable reimbursement for travel from European Society for Medical Oncology; receiving consulting and travel fees from Peptomyc, Kelun Pharmaceuticals/Klus Pharma, Ellipses Pharma, Molecular Partners, IONCTURA (including serving on the scientific advisory board); Consulting fees from Vall d'Hebron Institute of Oncology/Ministero De Empleo Y Seguridad Social, Chinese University of Hong Kong, Boxer Capital, LLC, Tang Advisors, LLC receiving research funding from Blueprint Medicines, Black Diamond Therapeutics, Merck Sharp & Dohme, Hummingbird, Yingli and Vall d'Hebron Institute of Oncology/Cancer Core Europe; and serving as investigator in clinical trials with Novartis, Spectrum Pharmaceuticals, Symphogen, BioAlta, Pfizer, GenMab, CytomX, Kelun‐Biotech, Takeda‐Millenium, GalxoSmithKline, Taiho, Roche Pharmaceuticals, Hummingbird, Yingli, Bycicle Therapeutics, Merus, Curis, Bayer, AadiBioscience, Nuvation, ForeBio, BioMed Valley Discoveries, Loxo Oncology, Hutchinson MediPharma, Cellestia, Deciphera, Ideaya, Amgen, Tango Therapeutics, Mirati Linnaeus Therapeutics, and Cancer Core Europe. DK reports NIH Grants (Clinical Translational Science Award 2019–2024; NCI Core Grant, Clinical Translational 2002–2023); Consulting, Black Beret Life Sciences; Advisory Board, Affigen, Phosplatin. FMB declares the following conflicts. Consulting: AbbVie, Aduro BioTech Inc., Alkermes, AstraZeneca, Daiichi Sankyo Co. Ltd., DebioPharm, Ecor1 Capital, eFFECTOR Therapeutics, F. Hoffman‐La Roche Ltd., GT Apeiron, Genentech Inc., Harbinger Health, IBM Watson, Infinity Pharmaceuticals, Jackson Laboratory, Kolon Life Science, Lengo Therapeutics, Menarini Group, OrigiMed, PACT Pharma, Parexel International, Pfizer Inc., Protai Bio Ltd, Samsung Bioepis, Seattle Genetics Inc., Tallac Therapeutics, Tyra Biosciences, Xencor, Zymeworks; Advisory Committee: Black Diamond, Biovica, Eisai, FogPharma, Immunomedics, Inflection Biosciences, Karyopharm Therapeutics, Loxo Oncology, Mersana Therapeutics, OnCusp Therapeutics, Puma Biotechnology Inc., Seattle Genetics, Sanofi, Silverback Therapeutics, Spectrum Pharmaceuticals, Zentalis; Sponsored Research (to MD Anderson Cancer Center): Aileron Therapeutics, Inc. AstraZeneca, Bayer Healthcare Pharmaceutical, Calithera Biosciences Inc., Curis Inc., CytomX Therapeutics Inc., Daiichi Sankyo Co. Ltd., Debiopharm International, eFFECTOR Therapeutics, Genentech Inc., Guardant Health Inc., Klus Pharma, Takeda Pharmaceutical, Novartis, Puma Biotechnology Inc., Taiho Pharmaceutical Co.; Honoraria: Chugai Biopharmaceuticals. Other (Travel Related) European Organization for Research and Treatment of Cancer (EORTC), European Society for Medical Oncology (ESMO). VS receives research funding for clinical trials from Novartis, Bayer, GlaxoSmithKline, Nanocarrier, Vegenics, Celgene, Northwest Biotherapeutics, Berghealth, Incyte, Fujifilm, Pharmamar, D3, Pfizer, Multivir, Amgen, Abbvie, Alfa‐sigma, Agensys, Boston Biomedical, Idera Pharma, Inhibrx, Exelixis, Blueprint medicines, Loxo oncology, Takeda and Roche/ Genentech, National Comprehensive Cancer Network, NCI‐CTEP and UT MD Anderson Cancer Center. Travel: Novartis, Pharmamar, ASCO, ESMO. SPP receives Clinical Trial Research Support/Grant Funding through the institution from the following sources: AbbVie, Inc.; ABM Therapeutics, Inc.; Acepodia, Inc; Alkermes; Aminex Therapeutics; Amphivena Therapeutics, Inc.; BioMarin Pharmaceutical, Inc; Boehringer Ingelheim; Bristol Myers Squib; Cerulean Pharma, Inc.; Chugai Pharmaceutical Co., Ltd; Curis, Inc.; Cyclacel Pharmaceuticals; Daiichi Sankyo; Eli Lilly; ENB Therapeutics; Epigenetix Inc.; Five Prime Therapeutics; F‐Star Beta Limited; F‐Star Therapeutics; Gene Quantum; Genmab A/S; Gilead Sciences, Inc.; GlaxoSmithKline; Helix BioPharma Corp.; Hengrui Pharmaceuticals, Co., Ltd.; HiberCell, Inc.; Immorna Biotherapeutics, Inc.; Immunomedics, Inc.; Incyte Corp.; Jacobio Pharmaceuticals Co., Ltd.; Jiangsu Simcere Pharmaceutical Co., Ltd.; Lytix Biopharma AS; Medimmune, LLC.; Medivation, Inc.; Merck Sharp and Dohme Corp.; Nectin Therapeutics, Ltd.; Novartis Pharmaceuticals; Pieris Pharmaceuticals, Inc.; Pfizer; Phanes Therapeutics; Principia Biopharma, Inc.; Puma Biotechnology, Inc.; Purinomia Biotech, Inc.; Rapt Therapeutics, Inc.; Replimune; Seattle Genetics; Silverback Therapeutics; Synlogic Therapeutics; Taiho Oncology; Tesaro, Inc.; TransThera Bio; ZielBio, Inc.; NCI/NIH; P30CA016672 – Core Grant (CCSG Shared Resources); consultancy: CRC Oncology. AN declares the following, Research funding from NCI, EMD Serono, MedImmune, Healios Onc. Nutrition, Atterocor/Millendo, Amplimmune, ARMO BioSciences, Karyopharm Therapeutics, Incyte, Novartis, Regeneron, Merck, Bristol Myers Squibb, Pfizer, CytomX Therapeutics, Neon Therapeutics, Calithera Biosciences, TopAlliance Biosciences, Eli Lilly, Kymab, PsiOxus, Arcus Biosciences, NeoImmuneTech, Immune‐Onc Therapeutics, Surface Oncology, Monopteros Therapeutics, BioNTech SE, Seven & Eight Biopharma, and SOTIO Biotech AG; Advisory board/Consulting fees from Deka Biosciences, NGM Bio, PsiOxus Therapeutics, Immune‐Onc Therapeutics, STCube Pharmaceuticals, OncoSec KEYNOTE‐695, Genome & Company, CytomX Therapeutics, Nouscom, Merck Sharp & Dohme Corp, OncoNano, Servier, Lynx Health, AbbVie, PsiOxus; Travel and accommodation expense from ARMO BioSciences, NeoImmuneTech, NGM Biopharmaceuticals; Honoraria for speaking engagements from AKH Inc, The Lynx Group, Society for Immunotherapy of Cancer (SITC), Korean Society of Medical Oncology (KSMO), Scripps Cancer Care Symposium, ASCO Direct Oncology Highlights, European Society for Medical Oncology (ESMO), CME Outfitters.

## ETHICAL APPROVAL AND CONSENT TO PARTICIPATE

Ethical approval was sought from Institutional Review Board (IRB) prior to commencing on study. For patient samples (such as tissue/blood samples), informed consent was obtained. The University of Texas MD Anderson Cancer Center Institutional Review Board approved this study.

## Supporting information


Data S1.

## Data Availability

The datasets used and/or analyzed during the current study are available from the corresponding author on reasonable request. The authors follow the FAIR principles (Findability, Accessibility, Interoperability, Reproducibility) for data access.
