# Supplementary material for: Phase I study of sapanisertib (CB‐228/TAK‐228/MLN0128) in combination with ziv‐aflibercept in patients with advanced solid tumors
Source: Cancer Med. 2024 Feb 24;13(3):e6877. doi: 10.1002/cam4.6877 (PMC10891443; doi:10.1002/cam4.6877)
Supplement: Supplementary file 1 — Data S1. [file CAM4-13-e6877-s001.docx]

**Supplementary Table 1. Planned Dose Escalation Treatment Schema**

| **Dose Escalation Schedule** | | |
| --- | --- | --- |
| **Dose Level** | **Dose*** | |
|  | **MLN0128 (TAK-228)**  **(mg PO for 3 days on and 4 days off) Starting on Cycle 1 Day 2** | **Ziv-Aflibercept (mg/kg IV Q 2 weeks)**  **Starting on Cycle 1 Day 1** |
| Level -1 | 3 | 2 |
| Level 1 | 4 | 2 |
| Level 2 | 4 | 3 |
| **Doses are stated as exact dose in units (*e.g.*, mg/m^2^, mcg/kg, etc.) rather than as a percentage.*  *As of Amendment 13/ Version 07, all newly enrolled patients will be given the milled formulation of MLN0128 (TAK-228), and a new dose escalation is being explored. Starting with Amendment 14/Version 08, the dose escalation will be re-started at Dose Level 1 with the updated dose level escalation.*  *Both study drugs will have a +/- 2 day window. If the ziv-aflibercept dosing is rescheduled, then the MLN0128 (TAK-228) dosing will be adjusted accordingly.* | | |

**Supplementary Table 2. Clinical details regarding 19 patients treated at dose level 2a, i.e. sapanisertib 4 mg QD po 3 days on and 4 Days off + 3 mg/kg Ziv-aflibercept, IV Q2W 28 day cycle.**

| **Dose Level** | **Tumor type** | **No. of Prior Systemic Therapies in Metastatic Setting** | **Age at c1d1** | **Days on Study** | **Months on study** | **Reason for Removal from Study** | **Best Response** | **Best Response % Change from baseline** | **Name of Molecular Profiling Test** | **Molecular Alteration** | **Comments** | **Toxicity (related, possible/ probable/ definite)** |
| --- | --- | --- | --- | --- | --- | --- | --- | --- | --- | --- | --- | --- |
| Dose level 2a | Renal Cell Carcinoma | 6 | 45 | 40.00 | 1.3 | Insurance issue | NE |  | 1) STGA-DNA 2018 2) Liquid Biopsy Panel V1 | 1) PTEN ,  2) PTEN | Patient was taken off treatment prior to  first restaging ;  insurance issues | Nil related |
| Dose level 2a | Cervical Cancer | 2 | 47 | 103.00 | 3.4 | PD | PR (unconfirmed) | -32 | STGAv1 | AKT1 (STGAv1) | PR followed by PD = SD | Nil related |
| Dose level 2a | Colorectal Cancer | 6 | 68 | 141.00 | 4.6 | PD | SD | -21 | STGAv1 | APC , PIK3CA , APC , KRAS |  | Grade 1 oral mucositis |
| Dose level 2a | Endometrial Sarcoma | 5 | 53 | 91.00 | 3.0 | PD | SD | -24 | 1) Guardant360 2) CARIS | 1) MET 2) KRAS , NOTCH1 , PIK3CA |  | Grade 1 nausea; grade 1 vomiting; grade 2 fatigue |
| Dose level 2a | Colorectal Cancer | 3 | 40 | 112.00 | 3.7 | fistula | PR (unconfirmed) | -73 | CMS50 | KRAS , TP53 | PR followed by no  evaluation = SD | Grade 1 oral mucositis |
| Dose level 2a | Sarcoma | 6 | 46 | 25.00 | 0.8 | PD | PD | 36 | STGA-DNA 2018 | TP53 , TSC2 |  | Nil related |
| Dose level 2a | Endometrial Cancer | 3 | 63 | 172.00 | 5.7 | PD | SD | 0 | STGA-DNA 2018 | PPP2R1A , TP53 |  | Grade 2 proteinuria |
| Dose level 2a | Vulvar Cancer | 4 | 59 | 141.00 | 4.6 | intolerability | SD | -23 | STGA-DNA 2018 | PIK3CA |  | Grade 3 vomiting; grade 2 diarrhea; grade 2 fatigue; grade 1 hypertension |
| Dose level 2a | Endometrial Cancer | 1 | 55 | 102.00 | 3.4 | PD | SD | -4 | STGA-DNA 2018 | TP53 , PPP2R1A , TSC2 |  | Grade 1 oral pain; grade 1 epistaxis; grade 1 nausea |
| Dose expansion 2a | Cervical Cancer | 6 | 69 | 68.00 | 2.2 | Toxicity | SD | -27 | FoundationOne  CMS50  FoundationOne CDx | KIT , PIK3CA , PTEN , PTEN , MED12 , TSC2 , ARID1B , FGF3 , FGF4 , NF1 , PRDM1 , PRKDC , TET2 , TSHR , PTCH1 , PTEN , TSC2 , BCORL1 , BTG1 , CREBBP , CREBBP , GRM3 , MED12 , MSH6 , DNMT3A , FGF3 , FGF4 , KLHL6 , MTOR , NF1 , NTRK1 , PIM3 , SPEN , TET2 , PRDM1 |  | Nil related |
| Dose expansion 2a | Merkle cell carcinoma | 1 | 80 | 176.00 | 5.8 | PD | SD | -4 | STGA-DNA 2018 | NF1 , MRE11A |  | Grade lipase increase (7 days) |
| Dose expansion 2a | Colorectal Cancer | 6 | 32 | 15.00 | 0.5 |  | NE |  | STGA-DNA 2018 | PTEN , TP53 , KRAS | Patient was taken off treatment prior to  first restaging | Nil related |
| Dose expansion 2a | Renal Cell Carcinoma | 4 | 32 | 59.00 | 1.9 | PD | CPD |  | STGA 2018 | No mutation | Pt death prior to  restaging scans | Grade 3 nausea |
| Dose expansion 2a | Melanoma | 6 | 41 | 181 | 5.9 | PD | PR (confirmed) | -40 | 1) STGA-DNA 2018 2) Tempus xT Assay | TSC1 , CDK4 , TSC1 , BAP1 ,  2) BAP1 , EPHA2 , ARID1B , MTOR PTPN11 |  | Nil related |
| Dose expansion 2a | Cervical Cancer | 4 | 59 | 124 | 4.1 | PD | SD | 0 | STGA 2018 CM50 KRAS | Inadequate DNA |  | Grade 1 oral pain, Grade 1 diarrhea |
| Dose expansion 2a | Colorectal Cancer | 4 | 58 | 57.00 | 1.9 | PD | PD | 20 | STGA-DNA 2018 | TP53 , MAP2K4 |  | Grade 1 oral mucositis; Grade 1 epistaxis |
| Dose expansion 2a | Head and Neck SSC | 3 | 65 | 67.00 | 2.2 | PD | PD | 5 | Solid Tumor Genomic Assay 2018 - RNA (Fusions) | Gene fusions none | SD for target lesion,  PD for non-target  lesion, PD overall | Grade 2 oral mucositis |
| Dose expansion 2a | Cancer of Unknown Primary, Other | 1 | 67 | 234.00 | 7.7 | PD | SD | 2 | STGA-DNA 2018 | BRAF , NTRK1 , NTRK2 , TP53 , CDKN2A , KIT , STK11, MDM2 , TERT, SMARCA4 , FANCD2 , POLE , ATM , NOTCH1 , ATM |  | Nil related |
| Dose expansion 2a | Leomyosarcoma | 5 | 48 | 630.00 | 20.7 | PD | PR (unconfirmed) | -30 | 1) unknown 2) STGA-DNA 2018 | 1) missense mutation at codon 246 in the p53 gene (g.14065 G>A; p.Met246Ile). TP53 |  | Grade 3 lipase increase; grade 1 anemia; grade 2 fatigue |

Abbreviations: SD, stable disease; PD, progression of disease; PR, partial response; NE, not evaluable; STGA-DNA 2018, Solid Tumor Genomics Assay 2018; CMS50, AmpliSeq sequencing panel (CMS50; Life Technologies)

**Supplementary Table 3. Molecular profile of each patient on study, including details of best response to treatment and molecular profiling details.**

| **Best Response** |  | **Sequencing platform** | **Alteration present** |
| --- | --- | --- | --- |
| PR (confirmed) | -63 | 1.Guardant 2.Foundation One | 1) None detected 2) TERT , CHD2 , CSF1R , GNAS , MAP3K1 , MLL , NOTCH1 , PTCH1 , SPEN , SPTA1 |
| SD | 15 | 1.Oncomine 2. FoundationOne | 1) Negative for mutation, fusion and amplification 2) ABL1 , ATRX , BRIP1 , MSH3 , MYST3, NSD1 , PDCD11 |
| SD | -8 | Information not available | Information not available |
| SD | No Target Lesion | NGS platform not named | PIK3CA |
| SD | -24 | 1.Guardant360 2.CARIS | 1.MET 2.KRAS, NOTCH1 , PIK3CA |
| SD | -16 | NCI MATCH | FBXW7 mut |
| SD | -8 | STGAv1 | TP53, BRCA1 |
| PR (unconfirmed) | -73 | CMS50 | KRAS, TP53 |
| SD | -26 | FoundationOne | PIK3CA , PIK3CA , CDH1 , EPHB1 , MLL2 , MLL2 , MLL3 , MLL3 , TERT , APC , ARID1B , ATR , CDH1 , CEBPA , DOT1L , DICER1 , EPHA7 , FANCL , GATA6 , KLHL6 , MLL , MLL3 , MSH2 , PDGFRB , POLE , RUNX1 , SMARCA4 , TBX3 |
| SD | 6 | Guardant360 | NF1, RB1 |
| NE |  | STGAv1 | TP53, BRCA2 |
| NE |  | STGA-DNA 2018 | PTEN, TP53 , KRAS |
| PD | 36 | STGA-DNA 2018 | TP53, TSC2 |
| CPD |  | STGA 2018 | No mutation |
| PR (confirmed) | -40 | 1.STGA-DNA 2018  2.Tempus xT Assay | 1.TSC1, CDK4 , TSC1 , BAP1 ,  2. BAP1 , EPHA2 , ARID1B , MTOR PTPN11 |
| SD | 0 | STGA 2018 CM50 KRAS | Insufficient DNA |
| SD | -23 | STGA-DNA 2018 | PIK3CA |
| PD | 20 | STGA-DNA 2018 | TP53, MAP2K4 |
| SD | -4 | STGA-DNA 2018 | TP53, PPP2R1A , TSC2 |
| PD | 5 | Solid Tumor Genomic Assay 2018 - RNA (Fusions) | None identified |
| SD | 0 | STGA-DNA 2018 | PPP2R1A, TP53 |
| SD | 2 | STGA-DNA 2018 | BRAF, NTRK1 , NTRK2 , TP53 , CDKN2A , KIT , STK11, MDM2 , TERT, SMARCA4 , FANCD2 , POLE , ATM , NOTCH1 , ATM |
| SD | -8 | CMS50 | None identified |
| PR (unconfirmed) | -30 | 1.NGS platform not named 2.STGA-DNA 2018) | 1. missense mutation at codon 246 in the p53 gene (g.14065 G>A; p.Met246Ile). 2.TP53 |
| PD | 46 | CMS50 | KIT , TP53 |
| SD | 14 | CMS50 | None identified |
| SD | -7 | Information not available | Information not available |
| SD | -3 | 1.Single Gene Assay) 2.STGAv1 | 1.KRAS , 2. APC, KRAS |
| SD | -8 | 1.CMS50 2.Guardant360) (Liquid Biopsy Panel V1) | 1.ATM, KIT  2. ATM , KIT , APC , BRAF, BRAF , MET , TP53 , BRCA2 , ESR1 , GATA3, MTOR , APC , HRAS , TP53 , ATM , ESR1 , BRCA2 |
| NE |  | 1.STGA-DNA 2018  2.Liquid Biopsy Panel V1) | 1.PTEN ,  2. PTEN |
| PD | 125 | CMS50) | TP53 |
| SD | No Target Lesion | 1.STGA-DNA 2018)  2.Liquid Biopsy Panel V1) | 1.SETD2 , CDK12, CDK12, 2.SETD2 , CDK12 , CDK12 , TP53 |
| SD | -23 | CMS50 | PIK3CA , TP53 |

Abbreviations: SD, stable disease; PD, progression of disease; PR, partial response; NE, not evaluable; STGA-DNA 2018, Solid Tumor Genomics Assay 2018; CMS50, AmpliSeq sequencing panel (CMS50; Life Technologies).

**Supplementary Table 4. Treatment related Adverse Events for Dose Expansion Cohort (n=10).**

| **Adverse Event** | **G1** | **G2** | **G3** | **Any grade** |
| --- | --- | --- | --- | --- |
| Total | 5 | 3 | 4 | 12 |
| Anemia |  | 1 |  | 1 |
| Diarrhea | 1 |  |  | 1 |
| Epistaxis | 1 |  |  | 1 |
| Fatigue |  | 1 |  | 1 |
| General disorders and administration site conditions - Other | 1 |  |  | 1 |
| Lipase increased |  |  | 2 | 2 |
| Mucositis oral | 1 | 1 |  | 2 |
| Nausea |  |  | 1 | 1 |
| Oral pain | 1 |  |  | 1 |
| Vomiting |  |  | 1 | 1 |
| White blood cell decreased |  |  |  | 0 |
